# The physical activity profiles of South Asian ethnic groups in England

**DOI:** 10.1136/jech-2015-206455

**Published:** 2015-12-16

**Authors:** Prachi Bhatnagar, Nick Townsend, Alison Shaw, Charlie Foster

**Affiliations:** 1 Nuffield Department of Population Health, British Heart Foundation Centre on Population Approaches for Non-Communicable Disease Prevention, University of Oxford, Oxford, UK; 2 Nuffield Department of Population Health, University of Oxford, Oxford, UK

**Keywords:** PHYSICAL ACTIVITY, EPIDEMIOLOGY, ETHNICITY, HEALTH BEHAVIOUR, Health inequalities

## Abstract

**Background:**

To identify what types of activity contribute to overall physical activity in South Asian ethnic groups and how these vary according to sex and age. We used the White British ethnic group as a comparison.

**Methods:**

Self-reported physical activity was measured in the Health Survey for England 1999 and 2004, a nationally representative, cross-sectional survey that boosted ethnic minority samples in these years. We merged the two survey years and analysed data from 19 476 adults. The proportions of total physical activity achieved through walking, housework, sports and DIY activity were calculated. We stratified by sex and age group and used analysis of variances to examine differences between ethnic groups, adjusted for the socioeconomic status.

**Results:**

There was a significant difference between ethnic groups for the contributions of all physical activity domains for those aged below 55 years, with the exception of walking. In women aged 16–34 years, there was no significant difference in the contribution of walking to total physical activity (p=0.38). In the 35–54 age group, Bangladeshi males have the highest proportion of total activity from walking (30%). In those aged over 55 years, the proportion of activity from sports was the lowest in all South Asian ethnic groups for both sexes.

**Conclusions:**

UK South Asians are more active in some ways that differ, by age and sex, from White British, but are similarly active in other ways. These results can be used to develop targeted population level interventions for increasing physical activity levels in adult UK South Asian populations.

## Introduction

Physical inactivity is associated with an increased risk of cardiovascular diseases, colon cancer, breast cancer, musculoskeletal disorders and depression.^1^ More than a third of the English adult population is insufficiently physically active to meet the UK recommendations^2^ of at least 150 min of moderate-intensity aerobic activity every week.

UK South Asians suffer from higher rates of cardiovascular disease and diabetes^3^; these groups are also known to perform less physical activity than the White British population and this is particularly true of some women from South Asian groups.^4^ Differences in physical activity prevalence between ethnic groups are most often attributed to cultural differences and socioeconomic factors.^5^ There is qualitative research indicating a preference for types of physical activity in UK South Asians,^6^ but there is little population-level information.

People can obtain sufficient physical activity through different domains, including walking, sports, housework, DIY and occupation. Previous research that investigated the relative contributions of different domains of active adults reported that younger active people tended to play more sports and older active people tended to do more walking.^7^ Policies to increase physical activity in the adult population need to be targeted appropriately, but there is currently little information about which types of activities ethnic minority groups are likely to partake in and how this differs by age.

Our study aimed to investigate types of leisure-time activities among people from Indian, Pakistani and Bangladeshi ethnic groups, using the White British as a comparison; we investigated sex and age differences.

## Method

This study used information from the Health Survey for England (HSE) 1999 and 2004, which is an annual, nationally representative cross-sectional survey. In these 2 years, the HSE boosted the ethnic minority sample; we therefore obtained a larger sample size by combining the two survey years, as has been done in previous research.^8^ There are ethnic minority participants in other years of the HSE, but the sample size is too small to allow analysis by subgroup. To combine the 2 survey years, we identified all variables in both years to be included in the final data set. We then prepared the data in each year for merging by ensuring that the definitions, categories and names of each variable in both years matched. The files were then merged using Stata V.11 to create a master data set.

The HSE physical activity questions were adapted from the Allied Dunbar National Fitness Survey^9^ and asked of all people aged over 16 years. All respondents were asked questions about their physical activity in the past 4 weeks. For heavy housework, heavy manual work, walking and sports, respondents were asked to recall the total number of days in the past 4 weeks on which they had done that particular activity for 30 min or more. Occupational physical activity was not included in this analysis as we aimed to describe leisure-time activities.

To identify ethnic groups in the household, initial screening involved asking the person whether anyone from a list of ethnic groups lived at the household. Once this was established, individual respondents were asked to confirm their ethnic background, by choosing from a predefined list with an option of ‘any other group’.

We grouped age into three categories, to retain a larger sample size when analysing by subgroup of age. The HSE provides an age variable grouped into 10-year age-bands, so we combined these to create age groups 16–34, 35–54 and 55 and above for this analysis. To measure occupational social class, participants answered a standard set of questions to assign them to the Registrar General Social classification system.

### Analysis

We created a total physical activity variable by combining the individual responses for the different domains of heavy housework, heavy manual work and gardening (we refer to this as DIY), walking and sports; this contained the total number of physical activity events for each person. An event was defined as any activity done lasting 30 min or more. Using this information, we created stacked bar-graphs for each ethnic group, by age and sex, to illustrate the different contributions of each physical activity domain to overall physical activity.

We then examined whether there were statistical differences in the proportions of physical activity domains between ethnic groups. To test for differences in physical activity types between ethnic groups, we used analysis of variance tests (ANOVAs) to explore whether there was a difference in the mean total number of physical events for total activity and for the proportion that each physical activity domain contributed to total physical activity. We used Bonferroni tests to explore how ethnic groups differed, for total physical activity and for each domain. We then added occupational social class into the ANOVA model to test the association of socioeconomic status with physical activity type independently of ethnicity.

## Results

The final data set contained 19 476 people. Table 1 describes the basic characteristics of the sample. South Asian ethnic groups had a younger age profile than did the White British group, with the majority being aged under 55 years.

**Table 1:** Characteristics of participants in final data set, Health Survey for England 1999 and 2004 (n=19 476)

	Indian	Pakistani	Bangladeshi	White
	n=2448	n=2185	n=1965	n=12 878
	Percentage	Percentage	Percentage	Percentage
Age group				
16–34	38	55	58	26
35–54	43	33	28	36
55+	19	12	14	38
Generation				
UK-born	26	31	17	95
Born abroad	74	69	83	5
Gender				
Men	48	48	47	44
Women	52	52	53	56
Social class				
Professional	7	3	1	5
Managerial technical	23	11	7	27
Skilled non-manual	21	15	11	25
Skilled manual	11	14	15	19
Semi-skilled manual	21	17	19	16
Unskilled manual	3	2	2	5
Full-time students	5	9	11	1
All other never worked	8	28	34	2

The mean number of physical activity events was consistently higher in the White British group, followed by the Indian, Pakistani then Bangladeshi ethnic groups; the total amount of activity declined with age (table 2). Men had a higher mean number of physical activity events than women in the 16–34 and 55 and above age groups. In the 35–54 age group, Pakistani men had a lower mean number of events than Pakistani women of the same age and Indian men and women in this age group had a similar mean number of events.

**Table 2:** Mean number of total physical activity events lasting 30 min or more in the past 4 weeks, by ethnic group, sex and age (n=19 476)

	MEN				WOMEN			
	16–34	35–54	55+	Total	16–34	35–54	55+	Total
Indian	11.6	7.5	5.7	8.6	9.0	7.6	2.2	7.2
Pakistani	10.0	4.7	2.7	7.2	7.5	6.3	2.4	6.5
Bangladeshi	8.5	5.1	1.9	6.3	5.4	3.6	0.9	4.5
White	15.2	10.7	6.9	10.5	11.0	10.5	5.8	8.8

The majority of people in all ethnic groups did not meet the pre-2011 recommended levels of physical activity. Overall, more men than women met the recommended levels of activity, which was consistent across ethnic and age groups. The lowest levels of physical activity were in the Pakistani and Bangladeshi groups, although for all South Asian women aged 55 years and above, more than 90% did not meet the recommended levels of physical activity.

### Contributions of physical activity domains to total physical activity

**Figure 1:**
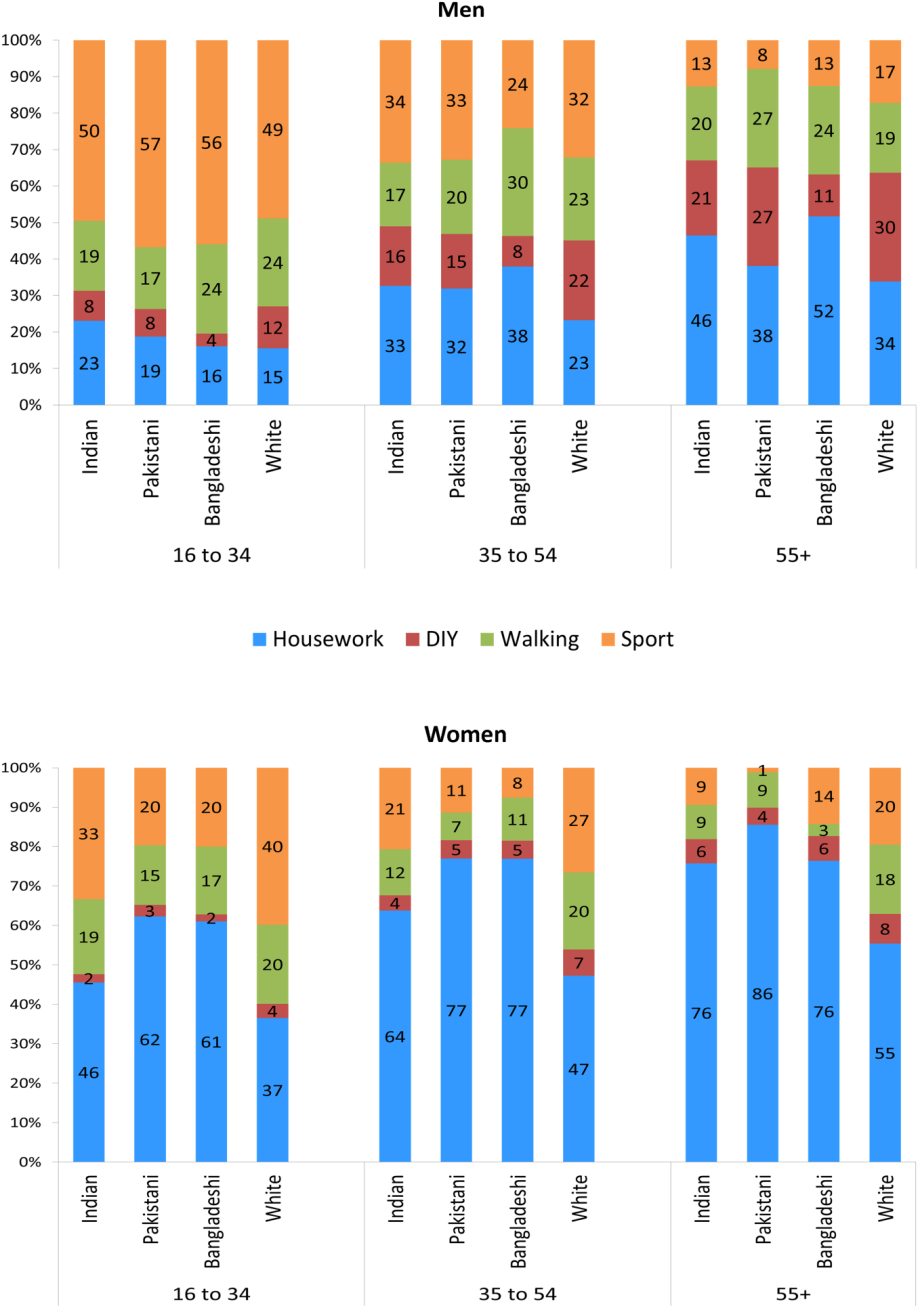
Percentage that each physical activity domain contributes to total physical activity, by sex, ethnic group and age group, Health Survey for England 1999 & 2004.

Figure 1 illustrates how each domain contributes to total activity for each ethnic group by age and sex. Differences between ethnic groups are apparent within all three age groups and tables 3 and 4 provide ANOVA results showing whether there was a statistical difference between ethnic groups after adjusting for occupational social class. The proportion of total activity from sports declined with age for all ethnic groups and the proportion of activity from housework increased with age. The contribution of housework to total physical activity was much higher in women compared to men in all ethnic groups and ages, although differences between the ethnic groups are apparent for both sexes. Within women, the South Asian groups appear to have a higher proportion of housework and a lower proportion of DIY contributing to physical activity compared to the White British group.

**Table 3:** Mean physical activity events* and percentage contribution of each domain to total physical activity in men, by ethnic group and age

	16–34		35–54		55+	
	Mean	Percentage	Mean	Percentage	Mean	Percentage
Housework						
Indian	1.3 (1.0 to 1.6)	23	1.9 (1.4 to 2.4)	33	1.6 (1.0 to 2.2)	46
Pakistani	1.0 (0.7 to 1.3)	19	1.0 (0.7 to 1.3)	32	0.5 (0.1 to 1.0)	38
Bangladeshi	0.7 (0.5 to 1.0)	16	1.1 (0.6 to 1.6)	38	0.5 (0.2 to 0.8)	52
White	1.5 (1.3 to 1.7)	15	1.7 (1.6 to 1.9)	23	1.5 (1.4 to 1.7)	34
*ANOVA p value*	<0.00	<0.00	<0.00	<0.00	<0.00	<0.00
DIY						
Indian	0.7 (0.4 to 1.0)	8	1.0 (0.7 to 1.4)	16	0.8 (0.4 to 1.1)	21
Pakistani	0.6 (0.3 to 0.8)	8	0.6 (0.3 to 1.0)	15	0.4 (0.1 to 0.7)	27
Bangladeshi	0.2 (0.1 to 0.3)	4	0.3 (0.1 to 0.6)	8	0.1 (0.0 to 0.2)	11
White	1.6 (1.3 to 1.9)	12	2.0 (1.8 to 2.2)	22	1.9 (1.7 to 2.1)	30
*ANOVA p value*	<0.00	<0.00	<0.00	<0.00	<0.00	<0.00
Walking						
Indian	2.7 (2.0 to 3.5)	19	1.8 (1.3 to 2.4)	17	2.0 (1.1 to 2.8)	20
Pakistani	2.4 (1.8 to 3.1)	17	1.3 (0.8 to 1.8)	20	1.2 (0.3 to 2.1)	27
Bangladeshi	3.1 (2.1 to 4.2)	24	2.5 (1.6 to 3.4)	30	1.7 (0.3 to 3.1)	24
White	4.7 (4.2 to 5.2)	24	3.1 (2.8 to 3.4)	23	2.1 (1.8 to 2.3)	19
* ANOVA p value*	<0.00	<0.00	<0.00	<0.00	0.32	0.47
Sports						
Indian	6.1 (5.1 to 7.0)	50	2.6 (2.1 to 3.2)	34	0.8 (0.3 to 1.3)	13
Pakistani	5.7 (4.9 to 6.6)	57	2.3 (1.6 to 3.1)	33	0.2 (0.0 to 0.4)	8
Bangladeshi	4.7 (3.8 to 5.5)	56	1.7 (0.9 to 2.5)	24	0.2 (0.0 to 0.3)	13
White	7.7 (7.1 to 8.2)	49	3.9 (3.6 to 4.3)	32	1.5 (1.3 to 1.7)	17
*ANOVA p value*	<0.00	0.01	<0.00	<0.00	<0.00	0.35

*In the past 4 weeks, lasting 30 min or more; 95% CIs for the mean are in brackets; ANOVA tested for difference between all ethnic groups and is adjusted for occupational social class.

ANOVA, analysis of variance.

**Table 4:** Mean physical activity events* and percentage contribution of each domain to total physical activity in women, by ethnic group and age

	16–34		35–54		55+	
	Mean	Percentage	Mean	Percentage	Mean	Percentage
Housework						
Indian	3.0 (2.4 to 3.5)	46	3.7 (3.1 to 4.4)	64	1.3 (0.8 to 1.9)	76
Pakistani	3.6 (3.1 to 4.2)	62	4.0 (3.2 to 4.8)	77	2.3 (1.0 to 3.6)	86
Bangladeshi	2.1 (1.7 to 2.6)	61	2.2 (1.5 to 2.9)	77	0.4 (0.1 to 0.7)	76
White	3.0 (2.8 to 3.3)	37	3.7 (3.5 to 4.0)	47	2.3 (2.1 to 2.5)	55
* ANOVA p value*	<0.00	<0.00	<0.00	<0.00	<0.00	<0.00
DIY						
Indian	0.2 (0.0 to 0.5)	2	0.3 (0.1 to 0.5)	4	0.1 (0.0 to 0.1)	6
Pakistani	0.1 (0.1 to 0.2)	3	0.5 (0.1 to 0.8)	5	0.1 (0.0 to 0.3)	4
Bangladeshi	0.1 (0.0 to 0.3)	2	0.2 (0.1 to 0.3)	5	0.1 (−0.1 to 0.4)	6
White	0.4 (0.3 to 0.4)	4	0.6 (0.5 to 0.7)	7	0.5 (0.4 to 0.6)	8
*ANOVA p value*	0.09	<0.00	0.02	0.01	<0.00	0.85
Walking						
Indian	2.7 (2.1 to 3.4)	19	1.8 (1.2 to 2.4)	12	0.7 (0.1 to 0.4)	9
Pakistani	1.7 (1.2 to 2.2)	15	0.8 (0.4 to 1.2)	7	0.5 (−0.2 to 1.2)	9
Bangladeshi	1.5 (1.1 to 2.0)	17	0.7 (0.2 to 1.2)	11	0.0 (0.0 to 0.1)	3
White	3.1 (2.8 to 3.5)	20	3.1 (2.8 to 3.4)	20	1.7 (1.5 to 1.9)	18
*ANOVA p value*	<0.00	0.38	<0.00	<0.00	0.06	0.09
Sports						
Indian	3.1 (2.5 to 3.7)	33	2.2 (1.6 to 2.7)	21	0.2 (0.0 to 0.5)	9
Pakistani	1.9 (1.4 to 2.5)	20	1.2 (0.6 to 1.7)	11	0.1 (0.0 to 0.2)	1
Bangladeshi	1.6 (1.1 to 2.1)	20	0.5 (0.2 to 0.9)	8	0.0 (0.0 to 0.1)	14
White	4.5 (4.2 to 4.8)	40	3.0 (2.8 to 3.2)	27	1.3 (1.1 to 1.4)	20
*ANOVA p value*	<0.00	<0.00	<0.00	0.01	0.01	0.04

*In the past 4 weeks, lasting 30 min or more; 95% CIs for the mean are in brackets; ANOVA tested for difference between all ethnic groups and is adjusted for occupational social class.

ANOVA, analysis of variance.

#### Ages 16–34

There was a significant difference between all ethnic groups aged 16 to 34 years in the proportions of physical activity domains contributing to total physical activity, with the exception of walking. For women in this age group, the unadjusted model showed very weak evidence of a difference between ethnic groups for the contribution of walking to total activity (p=0.06); however, after adjusting for social class, there was no evidence of a statistical difference between ethnic groups (p=0.38). Adjusting for social class removed the statistically significant difference for the mean number of DIY events in this age group, indicating that occupational social class explains some of the difference between ethnic groups for a mean number of DIY events (p≤0.00 before adjustment for social class). Bonferroni tests showed that Indian men and women in the 16–34 years age group showed no evidence of being significantly different from White British men and women for the proportion of total activity from walking. Pakistani men had sport as a statistically significant higher proportion of their total activity compared to White British men. For South Asian women, however, sport contributes a much lower proportion to physical activity than for White British women, with over 60% of total activity coming from housework in Pakistani and Bangladeshi women aged 16–34 years.

#### Ages 35–54

There was a significant difference between ethnic groups for all physical activity domains. Of all the ethnic groups in this age group, Bangladeshi males have the highest proportion of total activity from walking (30%). For women, 77% of total activity came from housework for the Pakistani and Bangladeshi women, compared to 47% in the White British group. Bonferroni tests showed that all three South Asian groups had a contribution of housework to total activity significantly different from the White British group, although the evidence was weaker for the men in the Indian ethnic group (p=0.05). The proportion of activity from walking, DIY and sports was highest in White British women. The proportion of activity from sports in the Indian women of this age group was more than double that of the Pakistani and Bangladeshi women.

#### Ages 55 and above

In those aged over 55 years, the proportion of activity from sports was the lowest in all South Asian ethnic groups for both sexes; it was particularly low for Pakistani women, and Bonferroni tests confirmed that Pakistani women in this age group were significantly different from White British women in the contribution of sport to total activity. South Asian men had a higher proportion of total activity from walking, but there was no evidence of a statistical difference between the ethnic groups after adjusting for occupational social class (p=0.47). South Asian men also had a higher proportion of total activity from housework than White British men, which was significantly different between the ethnic groups (p≤0.00). For sports, there was a significant difference in the mean number of events between ethnic groups, but not in its contribution to total activity. For women aged over 55 years, housework accounted for the majority of South Asian women's activity and in a much higher proportion compared to White British women in this age group. The contribution of walking to total activity was 18% for White British women, compared to half of this for Indian and Pakistani women, and only 3% for Bangladeshi women; however, these differences were no longer statistically significant after adjusting for occupational social class.

## Discussion

### Summary of findings

We analysed 19 476 participants to produce the first physical activity domain profiles by ethnic group. We show that the mean number of total physical activity events differs between Indian, Pakistani, Bangladeshi and White British ethnic groups, and that this total activity comprises different types of physical activity.

Figure 1 and tables 3 and 4 demonstrate clearly that the types of physical activity undertaken by people in England vary according to their ethnic group, sex and age group. This analysis also highlights some similarities between ethnic groups; for example, in the younger Indian group, walking contributes a similar proportion to physical activity as in the majority White British group.

### Comparison to the existing literature

Bélanger *et al*^7^ examined age-related differences in physical activity types in the general population using the HSE 2008. This paper showed that the proportion of activity that comes from sports and exercise and fitness declines with age, and is particularly low in those aged above 45 years. This pattern was also apparent for each ethnic group in our study, with the proportion of activity from sports consistently lower in those aged over 55 years. Bélanger *et al* also found that occupational physical activity is a large contributor to total activity in men aged below 65 years. Since occupational activity has been excluded from the analysis in our study, it is possible that differences between men and women of different ethnic groups are partially accounted for by occupational physical activity, although controlling for occupational social class may have partially reduced this bias.

Although we could find no UK studies that had examined all types of physical activity undertaken by ethnic minorities, there are some studies published in the USA. An empirical study based in the USA showed that socioeconomic status explained much of the difference in the amount of leisure-time physical activity between Hispanics and non-white Hispanics, indicating that cultural differences are not always responsible for differences in physical activity behaviour between ethnic groups.^10^ Kandula and Lauderdale^11^ reported that immigrant Asian Americans were less likely to participate in leisure-time physical activity compared to American-born non-Asians; ‘Asian American’ in their study refers to Chinese and South Asian groups. Although our paper did not differentiate by generation, the majority of those who were UK-born were under the age of 35 years.

We were unable to investigate further the types of sports practised by different ethnic groups, but the Active People Survey indicates that there are cultural preferences for certain types of leisure-time physical activities in ethnic minority groups in England. Asian people are more likely to have participated in cricket and the gym, and weight-training and basketball are all popular among ethnic minority communities in the UK.^12^ South Asian women aged 16–34 years all play less sports as a proportion of total activity as compared to White British women of the same age; it is possible that South Asian women do not have as much access as women in the White British population to the sports they may prefer, such as those mentioned. Since ethnic groups tend to cluster in geographical areas,^13^ it is also possible that local facilities influence the types or amounts of sports that ethnic minorities in England play.

### Strengths and limitations

To the best of our knowledge, this is the first study to assess at a population level the different types of physical activities undertaken by South Asian groups in the UK. By combining two large nationally representative data sets, we have been able to analyse ethnic groups separately by sex and age. Many studies combine Indians, Pakistanis and Bangladeshis as ‘South Asian’ for analysis in order to boost the sample size and thus the power of the results; however, this results in a limited ability to usefully interpret the results. In the UK, the socioeconomic profiles of Indians, Pakistanis and Bangladeshis are quite different, as are their main religious identities. Both these factors have a high potential to affect physical activity behaviour, and we have shown that there are differences in the physical activity behaviour of these three ethnic groups, only some of which are explained by occupational social class.

Some main limitations of this study are rooted in the nature of the HSE 1999 and 2004 surveys. First, the data come from studies published in 1999 and 2004 and the physical activity profiles of ethnic groups could have changed over the past decade. Second, the physical activity questions included in the 1999 and 2004 HSE surveys were limited in their scope, offering only a self-reported measure for physical activity, which may introduce recall bias. The nature of the question ‘how many days in the past 4 weeks have you done (insert activity name here) for 30 min or more?’ does not allow for an accurate calculation of the number of minutes of moderate to vigorous physical activity, as the more recent physical activity surveys do. There is also a possibility of misclassification bias in the chance of there being systematic differences between ethnic minorities in how they self-report physical activity; it is difficult to gain population-level information on this; however, there is some evidence that South Asians may under-report their total levels of activity.^14^

Studies done in other populations^7^ ^15^ were able to use more domains of physical activity, but in this study only the four broad domains of housework, DIY, walking and sports could be included for analysis. Ideally, ‘sports’ could have been broken down into types of sport, such as in the paper by Bélanger *et al*, and ‘walking’ could have been broken down into ‘walking for leisure’ and ‘active transport’. Occupational physical activity was not included in this study, which could affect the proportions of physical activity types contributing to total physical activity. However, the inclusion of occupational social class in the regression analyses should go some way towards assessing the contribution of manual work towards total physical activity.

Owing to sample size limitations, we were unable to stratify the results by occupational social class in addition to ethnic group, sex and age, or adjust for other socioeconomic variables. We would recommend that future research explore how much of the differences between ethnic groups can be explained by socioeconomic status and environmental factors relating to deprivation.

## Conclusions

This analysis shows that while South Asian ethnic minority groups in England are active in different ways from the White British population, and from each other, there are also some similarities.

It is important to understand the different ways in which ethnic groups are active, as this allows physical activity interventions to be tailored appropriately, while in the cases where activity patterns are similar to the majority population, tailored interventions may be unnecessary. Activity patterns change with age, as has been shown in the general population, indicating that age-appropriate interventions are necessary for all South Asian groups. It is also possible that some of these age differences are due to generational status, with UK-born ethnic groups having very different childhood experiences from ethnic groups who were born in other countries. This analysis, however, cannot provide detailed information on why age differences in physical activity patterns exist. It is likely to be due to factors that change throughout the life course, such as health, income and leisure-time, but it may also be due to differences in early childhood experiences, which stay with people throughout life.

Understanding the role of individual social class in physical activity patterns is also important, especially as some ethnic groups are mainly in the lower social classes. Social class had some impact on physical activity patterns, but it is difficult to know whether these differences are due to occupation, income or education levels. As some South Asian ethnic groups often live in deprived areas, and people in lower social classes frequently live in deprived areas, future research should explore whether local facilities and resources or individual socioeconomic status factors contribute to differences in physical activity profiles between South Asian ethnic groups.

**What is already known on this subject**

-  Physical inactivity is a risk factor for cardiovascular diseases, some cancers and musculoskeletal disorders.
- UK South Asians are less physically active than the White British population.

**What this study adds**

- We used a nationally representative survey to show that physical activity patterns differ between Indian, Pakistani and Bangladeshi ethnic groups in England. At all ages, sports contributes a lower proportion to total activity in Indian, Pakistani and Bangladeshi women compared to White British women, but Indian women aged 16–34 years have a similar proportion of total activity from walking as White British women in the same age group. We recommend that further research should be done on the types of physical activities available to and enjoyed by UK South Asian communities.
